# Supplementary material for: Water-immersion finger-wrinkling improves grip efficiency in handling wet objects
Source: PLoS One. 2021 Jul 21;16(7):e0253185. doi: 10.1371/journal.pone.0253185 (PMC8294484; doi:10.1371/journal.pone.0253185)
Supplement: Attachment — Submitted filename: GripLoadRatio_LogMeanValues.pdf [file pone.0253185.s002.pdf]

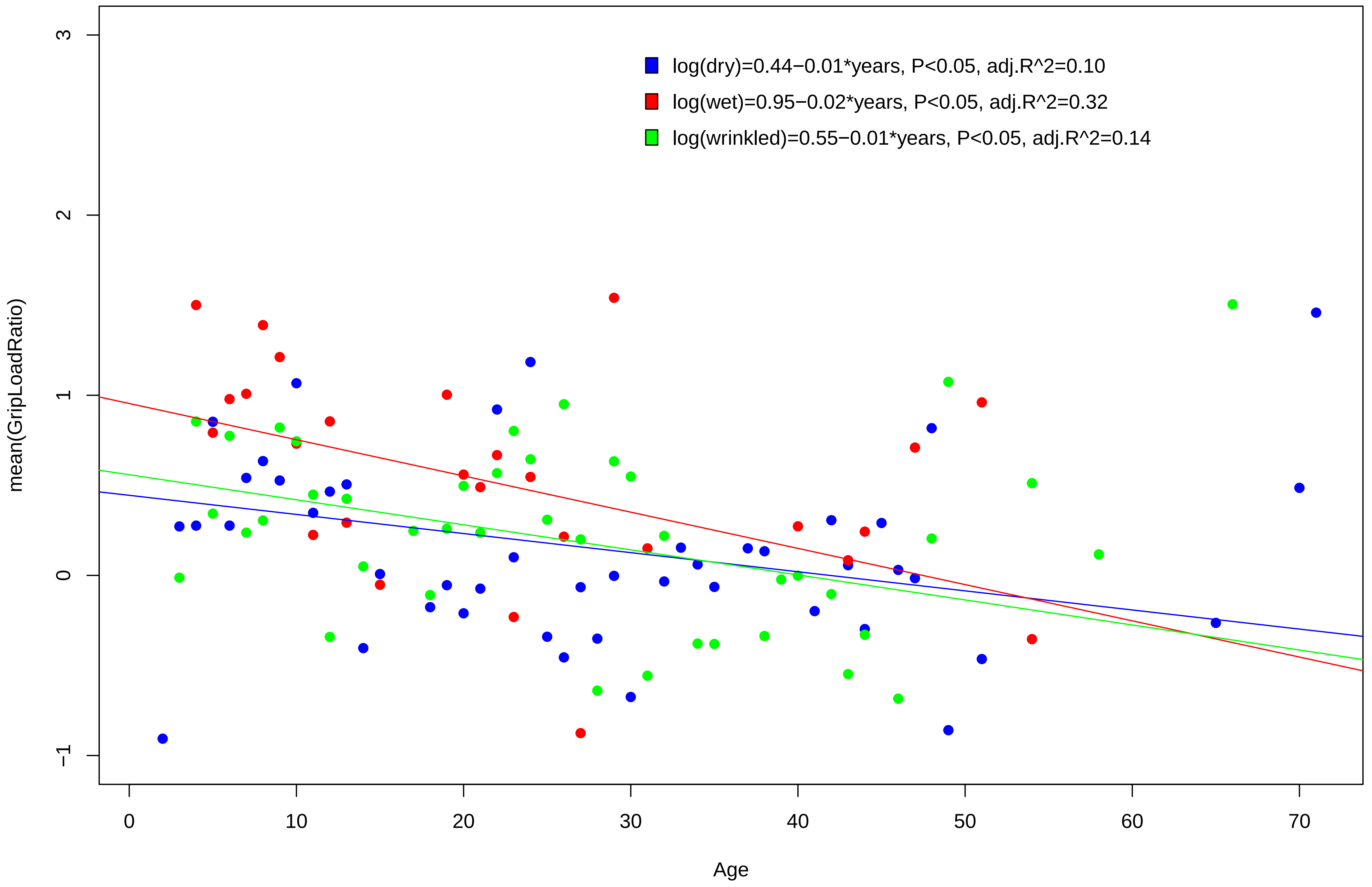

log(dry)=0.44−0.01*years, P<0.05, adj.R^2=0.10
log(wet)=0.95−0.02*years, P<0.05, adj.R^2=0.32
log(wrinkled)=0.55−0.01*years, P<0.05, adj.R^2=0.14
mean(GripLoadRatio)
Age
−1
0
1
2
3
0
10
20
30
40
50
60
70
