# Supplementary material for: Water-immersion finger-wrinkling improves grip efficiency in handling wet objects
Source: PLoS One. 2021 Jul 21;16(7):e0253185. doi: 10.1371/journal.pone.0253185 (PMC8294484; doi:10.1371/journal.pone.0253185)
Supplement: Attachment — Submitted filename: Wrinkles - Response - 14Apr21.docx [file pone.0253185.s001.docx]

**Water-immersion finger-wrinkling improves grip efficiency in handling wet objects**

**Nick J. Davis**

Dear Editor,

**Response to reviewers**

I am grateful to you and both reviewers for the time spent in reading and commenting on this manuscript. I am sure that we are all working under the same constraints, and so I am especially grateful for their careful attention during this difficult time in our history.

Below I have responded to each point raised by Reviewer #1. Reviewer #2 did not suggest any changes, but did make some helpful suggestions about further work. I hope the reviewers find the responses and the manuscript updates helpful.

Again I would like to thank you and the reviewers for your time and attention, and I hope you are all staying safe.

Nick.

**Reviewer #1**

The reviewer makes some excellent points, which are both detailed and thoughtful. I am very grateful for these comments, which I think have led to an improved manuscript. Where I have made additions to the manuscript in response to Reviewer #1’s comments, I have highlighted these in yellow in the revised manuscript. Note that I have also updated the analysis script that is filed with the data in the online repository.

*1) The research question(s) is not precisely stated nor justified.*

I agree that the research question is not completely clear. In the final paragraph of the Introduction I have clarified the purpose of the experiment. I believe that the experiment is well-justified in the preceding paragraphs, where I highlight the state of existing knowledge about wrinkled fingers, and touch on the relevant findings in grip- and load-force coordination. I had also planned for this work to speak to the position of skilled motor action in the evolution of the human lineage (as suggested in the Changizi “rain treads” paper), however I now realise that the supporting evidence on finger-wrinkles in non-human relatives is not available.

*2) The paper looks at a very heterogeneous age group. Considering the data provided by the author, the range is 2 - 71 years. This is neither in anatomical-physiological nor in psychological-cognitive sense a uniform group from whose behaviour one can draw general conclusions. Considering the age range, the assumption of a linear relationship between grip/load ratio and age is worth considering.*

I agree that the data here covers a wide range of ages, as well as other relevant demographics (as acknowledged in the Discussion). It is certainly true that grip- and load-force coordination changes through the lifespan, and I also touched on this (briefly) in the Discussion. The reviewer makes the very good point that we might not expect a linear relationship to hold between these measures when looking at such a diverse cohort. However I did not have any reason to hypothesise any other form of relationship either, so I planned to use the simplest possible analysis to capture what I suspected would be the linear change in the middle years. It turns out that the age effects were rather small, and not very surprising (young children were worse, but not much worse). However I included the analysis for completeness. This analysis motivates future work to look at developmental differences in wrinkly-finger object manipulation, where the age variable might become categorical.

*3) Why were characteristics of touch sensitivity not measured? There is psycho-physical and anatomical evidence for age-related differences.*

This is an excellent point, and I agree entirely. The busy environment of the Science Museum meant that a bit of experimental care had to be sacrificed in favour of creating an engaging experience for museum visitors. I plan to conduct follow-up experiments in the lab that would afford this sort of measurement. I hope I have been open in the manuscript about the constraints of the public environment. I have added to the ‘limitations’ section of the Discussion to highlight that static and dynamic touch perception was not measured.

My stint at the Science Museum coincided with a primary and secondary school vacation. I was told that on my busiest day at the museum, 21,000 people passed through the doors. Not all of these people visited my area, but it did make for a very busy few weeks of data collection. If I were lucky enough to do something like this again, I would plan better for an experience that required less hands-on supervision from me, and more self-directed tasks for the visitors.

*4) The description of the methodology is insufficient. Which statistical tests were used, which post-hoc tests, which correction procedures for unequal numbers of participants (e.g. Dry=231, Wet=72, Wrinkly=211)?*

The primary statistical tests are described in the “Data analysis” section of the Methods, and I believe I have described all primary tests here. I am very grateful that the reviewer asked about the unequal group sizes, as it had not occurred to me to test. I have now used Bartlett’s test to test for homogeneity of variance in the Anovas. Only one test failed this assumption, so a Kruskal-Wallis test was used instead. Post-hoc tests used Matlab’s ‘multcompare’ function, which uses the Tukey-Cramer HSD correction after an Anova, or mean ranks after the Kruskal-Wallis test. I have added this information to the Methods section, under “Data analysis”.

*The description of the figures is insufficient, the legends are incomplete and contain commented parts that belong in the discussion. What is the meaning of the coloured areas in Fig. 2? How was the average calculated? Is it the mean of all valid trials or is it the mean of the subjects' individual averages? In the latter case, how was intra-individual variability considered when calculating the averages (see age distribution)?*

The description of the shaded area was not clear. It represents the ±1 standard error of the mean grip force at each timepoint in the static hold phase. To calculate this, each participant’s mean grip force was calculated, then the means of these means were calculated to form the traces that are illustrated. The SEM is shown for the static phase only, as this is the part that most directly answers the question I started with. The caption for Figure 2 has been updated to reflect this, and I have added an indication of the three phases of the trial (attack, static, decay).

Intra-individual variance was not analysed in this study, as each person only contributed eight trials. Individual trials were excluded if certain criteria were not met, and individual participants’ data were only included if they made five successful trials. So the analysis only proceeded on ‘valid’ trials. These criteria are stated in the “Data analysis” section of the Methods, and operate automatically within the Matlab analysis script.

*In which interval were the data for Fig. 4 calculated? What does the inter-individual variability look like?*

This information was missing from the manuscript. It represents the entire trace, minus the first 1000 ms. I have added this information to the caption of Figure 4. This figure is essentially the same as Figure 2, and the variance for the grip trace is shown in Figure 2 for the static phase. I attempted to redraw Figure 4 with the variance illustrated but it turned out to be an unhelpful mess. My intention was for this to look something like a phase plot, where the key information is the principal trajectory through phase space. I don’t think this figure quite achieves that level of helpfulness, however it does show the grip safety margin quite neatly, so I believe it is worth retaining.

**Reviewer #2**

Reviewer #2 made some generous and positive comments about the manuscript, for which I am very grateful. The reviewer suggests that a within-subjects version of the experiment would be interesting. I entirely agree, especially as grip force strategies tend to be fairly stable within a participant (so revealing differences between conditions). I plan to pursue this as soon as in-person experiments are possible in my region. Again, I thank the reviewer for the heartwarming comments.
