# Supplementary material for: Water-immersion finger-wrinkling improves grip efficiency in handling wet objects
Source: PLoS One. 2021 Jul 21;16(7):e0253185. doi: 10.1371/journal.pone.0253185 (PMC8294484; doi:10.1371/journal.pone.0253185)
Supplement: Attachment — Submitted filename: Wrinkles - Response - 26May21.docx [file pone.0253185.s003.docx]

**Water-immersion finger-wrinkling improves grip efficiency in handling wet objects**

**Nick J. Davis**

Dear Editor,

**Response to reviewers (R2)**

Once again I am grateful for your and the reviewers’ time and attention. Both reviewers stated that they did not need to see the manuscript again, however Reviewer #1 (rather gratifyingly) used the data I uploaded, to suggest an additional analysis. I have done this analysis, and have added a short amount of text to this version of the manuscript.

Briefly, the suggestion was to disaggregate the different conditions (dry, wet, wrinkly) before regressing grip:load ratio against age. Reviewer #2 pointed out a possible nonlinear shape some of these regressions. I ran a similar analysis (using all data, which the reviewer did not do), and found qualitatively similar results. I have added a short amount of text to the Results section and to the Discussion that deals with this nonlinearity, and added one of the works cited in the previous review. As this additional analysis was exploratory I have not dwelt on it, but have acknowledged that there is some interesting work in this area.

Thank you again for your time and for the helpful suggestions.

Best wishes,

Nick.
